# Immediate Loading of Implant-Supported Single Crowns after Conventional and Ultrasonic Implant Site Preparation: A Multicenter Randomized Controlled Clinical Trial

**DOI:** 10.1155/2018/6817154

**Published:** 2018-08-14

**Authors:** Claudio Stacchi, Teresa Lombardi, Domenico Baldi, Calogero Bugea, Antonio Rapani, Giuseppe Perinetti, Angelo Itri, David Carpita, Guido Audenino, Giuseppe Bianco, Simone Verardi, Stefano Carossa, Gianmario Schierano

**Affiliations:** ^1^Department of Medical, Surgical and Health Sciences, University of Trieste, Italy; ^2^Private Practice, Cassano allo Ionio (CS), Italy; ^3^Department of Surgical Sciences, University of Genoa, Italy; ^4^Private Practice, Sestri Levante (GE), Italy; ^5^Private Practice, Calci (PI), Italy; ^6^CIR Dental School, Department of Surgical Sciences, University of Torino, Italy; ^7^Private Practice, Roma, Italy; ^8^Department of Periodontics, University of Washington, Seattle, WA, USA

## Abstract

**Aim:**

To compare implant survival rate and marginal bone loss (MBL) of immediately loaded single implants inserted by using ultrasonic implant site preparation (UISP) (test) and conventional rotary instrumentation (control).

**Methods:**

Two single implants were inserted for each patient: after randomization, test site was prepared by using an ultrasonic device (Piezosurgery Touch, Mectron, Italy) and control site was prepared by using the drills of the selected implant system (Premium AZT, Sweden & Martina, Italy), until reaching a final diameter of 3 mm in both groups. Identical implants (3.8x11.5 mm) were inserted in all sites at crestal level. Impressions were taken and screwed resin single crowns with platform-switched provisional abutments were delivered with 48 hours. Periapical radiographs were taken at provisional crown insertion (T0), 6 months (T1) and one year (T2) after prosthetic loading to measure MBL. All data were tested for normality and subsequently analyzed by paired samples t-test and forward multiple linear regression.

**Results:**

Forty-eight patients were treated in six centers with the insertion of ninety-six implants (48 test; 48 control). Four implants in four patients failed within the first six months of healing (two in test group; two in control group; no difference between groups). Forty patients (age 60.1±10.7 years; 22 female, 18 male) were included in the final analysis. Mean MBL after six months of loading was 1.39±1.03 mm in the test group and 1.42±1.16 mm in the control group (p>0.05) and after one year was 1.92±1.14 mm and 2.14±1.55 mm in test and control, respectively (p>0.05).

**Conclusions:**

No differences in survival rate and MBL were demonstrated between UISP and conventional site preparation with rotary instruments in immediately loaded dental implants: UISP, with its characteristics of enhanced surgical control and safety in proximity of delicate structures, may be used as a reliable alternative to the traditional drilling systems.

## 1. Introduction

Implant-supported restoration is currently considered as a predictable treatment option for single tooth loss, showing high success rate after 5 years [1]. The original two-stage procedure with delayed implant loading [2, 3] has been modified over the years with the introduction of early and immediate loading protocols, in the attempt to reduce treatment time and patient discomfort [4, 5]. According to recent studies, immediately and conventionally loaded implant-supported single crowns showed equally successful clinical outcomes regarding implant survival rate and marginal bone loss [6, 7]; moreover, patient's oral health-related quality of life was demonstrated to improve significantly after the application of immediately loaded implant-supported fixed prosthesis [8, 9]. However, an accurate presurgical planning and strict adherence to validated protocols are necessary to obtain optimal functional and aesthetic results when approaching these advanced techniques [10–12].

An adequate primary stability is the main prerequisite to apply an immediate loading protocol; a secure mechanical retention of the implant into the host bone is necessary to prevent detrimental micromovements which could lead to a failure of the osseointegration process [13–15]. There is no universal consensus about the minimum primary stability threshold to reach for a safe application of immediate loading protocols; however implant stability quotients (ISQ) >60-65 or peak insertion torques >35 Ncm are mostly accepted as minimum values [6, 16]. Moreover, it should be considered that primary stability decreases during the first month after implant insertion due to peri-implant bone remodeling following surgical trauma [17]: modifications of implant microgeometry have been introduced to enhance and accelerate bone healing response and limit this problem [18, 19].

Piezoelectric bone surgery has been introduced into clinical practice as an alternative possibility of performing osteotomies by using ultrasonic surgical systems [20]. In the last twenty years, various clinical applications of ultrasonic bone cutting in oral and maxillofacial surgery were widely investigated, obtaining promising results in terms of surgical control, precision, and safety [21–29].

Ultrasonic implant site preparation (UISP) has been analyzed in biomolecular and histologic animal studies, showing evidence of favourable bone healing response in the early period after implant insertion [30, 31]. Clinical studies showed that, if compared to the traditional drilling technique, UISP resulted in limited decrease of implant primary stability and in an earlier transition towards an increasing stability pattern, representing a potential additional benefit in immediate loading protocols [32–34].

The objective of this parallel-group, superiority randomized clinical trial (RCT) was to compare the clinical outcomes of immediately loaded single implants inserted by using two different techniques: UISP as test and rotary instruments as control.

## 2. Materials and Methods

### 2.1. Study Design

The present study was designed as a multicenter randomized controlled clinical trial, following CONSORT guidelines, and was conducted in six clinical centers in accordance with the Good Clinical Practice Guidelines (GCPs) and with the recommendations of the Declaration of Helsinki as revised in Fortaleza (2013) for investigations with human subjects. The study protocol had been authorized by Azienda Ospedaliero Universitaria “Città della Salute e della Scienza”, Torino, Italy, and recorded in a public register of clinical studies (www.clinicaltrials.gov, n°  NCT03357406). A meeting had been held among all the clinical centers before the beginning of the study, in order to illustrate surgical and prosthetic protocols and ensure that clinical operators applied a standardized approach. One clinical operator for each center received written instructions regarding the assessment of experimental parameters in order to obtain acceptable interexaminer consistency in data collection.

Prior to enrollment, all patients were asked to sign an informed consent form to document that they understood the aims of the study (including procedures, follow-up evaluations, and any potential risk involved). Patients were allowed to ask questions pertaining to this study and were thoroughly informed of alternative treatments.

This superiority trial tested the null hypothesis of no differences in survival rate and marginal bone loss between UISP (test group) and conventional site preparation with rotary instruments (control group) in immediately loaded dental implants.

### 2.2. Study Population

Eligible participants were all adult patients (age ≥18 years), needing two implant-supported single crowns with immediate loading in the upper or lower arch (in incisor, canine, or premolar area), based on accurate diagnosis and treatment planning. Patients underwent clinical examination to evaluate periodontal and occlusal conditions, and bone volume in the areas of interest was analyzed basing on cone-beam computed tomography.

Inclusion criteria were the following:

- healed bone crest (at least six months elapsed after tooth loss)
- residual bone crest with minimum width of 6 mm and minimum height of 13 mm
- both implant sites inserted in similar bone quality (i.e., adjacent or contralateral teeth)
- peak insertion torque comprised between 35 and 60 Ncm
- patient willing to and fully capable of complying with the study protocol
- written informed consent given

Exclusion criteria were the following:

- acute myocardial infarction within the past 2 months
- uncontrolled coagulation disorders
- poorly controlled diabetes (HBA1c > 7.5%)
- radiotherapy to the head/neck district within the past 24 months
- immunocompromised patient (HIV infection or chemotherapy within the past 5 years)
- present or past treatment with intravenous bisphosphonates
- psychological or psychiatric problems
- alcohol or drugs abuse
- full mouth plaque score and/or full mouth bleeding score >20%

### 2.3. Surgical Procedures

**Figure 1:**
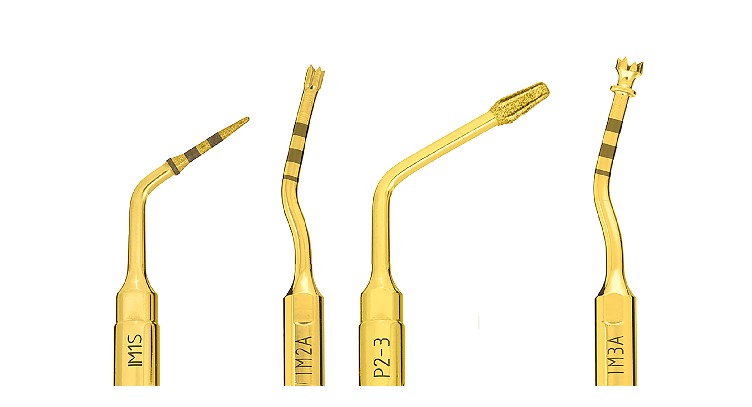
Sequence of ultrasonic inserts used for implant site preparation in the test group (final diameter 3.0 mm).

**Figure 2:**
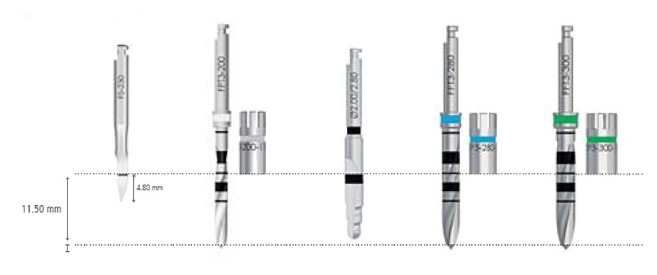
Sequence of rotary instruments used for implant site preparation in control group (final diameter 3.0 mm).

**Figure 3:**
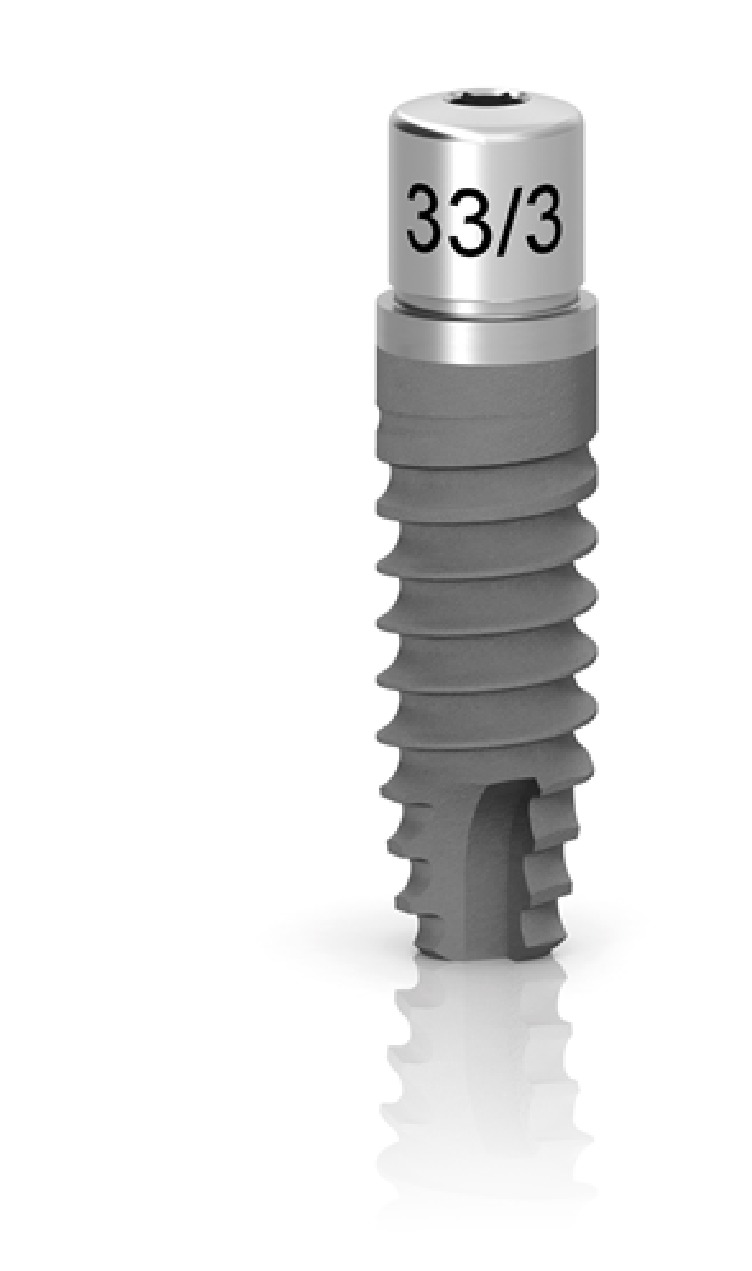
Internal hex implants with a sandblasted/etched surface (Premium AZT, Sweden & Martina, Italy), measuring 3.8x11.5 mm, were inserted in all sites at crestal level with healing abutment of reduced diameter (3.3 mm).

After performing anaesthesia (articaine 4% with epinephrine 1:100.000, Artin, Omnia, Italy) and raising a minimally invasive flap, the randomization sealed opaque envelope was opened by a clinical assistant and the surgeon was advised on the location of test and control sites. The preparation of the test site was performed by using an ultrasonic device (Piezosurgery Touch, Mectron, Italy) and the control site was prepared by using the drills of the selected implant system, following in both cases the sequence recommended by the manufacturer until a final diameter of 3 mm was reached in both groups (Figures 1 and 2). Internal hex implants with a sandblasted/etched surface (Premium AZT, Sweden & Martina, Italy), measuring 3.8x11.5 mm, were inserted in all sites at crestal level with healing abutment of reduced diameter (3.3 mm) (Figure 3). In case of adjacent implants, a minimal distance of 3 mm was respected between the two fixtures.

A clinical assistant recorded the peak insertion torque for both implants and the duration of the implant insertion procedure (time elapsed from the first cortical perforation to the complete insertion of the implant in the final position) for both techniques.

After suturing, a polyether impression (Impregum, 3 M Espe, USA) was performed by using an open tray and pick-up copings. Provisional restorations (screwed resin single crowns with platform-switched provisional abutments) were delivered with 48 hours, applying a nonfunctional loading.

Patients were prescribed with antibiotics for 6 days (amoxicillin 1 g twice a day or, in allergic patients, clarithromycin 250 mg twice a day) and nonsteroidal anti-inflammatory drug (ibuprofen 600 mg), when needed. Sutures were removed after 7 days. Definitive screwed metal ceramic crowns were delivered after 6 months of healing.

Periapical radiographs were performed with long cone paralleling technique using a film holding device, customized for each patient with a polyvinylsiloxane jig. Marginal bone level was assessed using a measuring software (Image J, National Institutes of Health, USA) by a single blinded and calibrated examiner (AR). All measurements were repeated three times at three different time points as suggested by Gomez-Roman and Launer [35] and each radiograph was calibrated using the known thread pitch of the implant as a reference. Examiner calibration was performed by assessing 20 radiographs, with another author (CS) who served as “reference examiner”. Intraexaminer and interexaminer concordances were 92.4% and 88.5%, respectively, for linear measurements within ±0.1 mm. The linear distance from the abutment/implant junction to the first bone contact was measured on mesial and distal aspect of the implant at provisional crown insertion (T0), 6 months (T1), one year (T2), and two years (T3) of prosthetic loading. Marginal bone loss (MBL) was defined as the difference among T0 and follow-up measurements (mean value between mesial and distal measurements was considered for each implant).

### 2.4. Outcomes

This study evaluated the following outcome measures:

- MBL: marginal bone loss at T1 and T2, using T0 as a reference
- Implant failure: implant mobility and/or any situation suggesting implant removal
- Biological and mechanical complications: any complication defined as an unexpected deviation from the normal treatment outcome, both biological (e.g., mucositis, peri-implantitis) and mechanical (e.g., implant fracture, prosthesis fracture, fixation screw loosening, etc.)

### 2.5. Sample Size and Statistical Power

The calculation was performed to detect a significant difference between the groups in marginal bone loss at 12 months of at least 0.2 mm with an expected standard deviation of 0.4 mm. Based on these data, a sample of 34 patients (68 implants; 34 test and 34 control cases) was needed to reach 80% of statistical power with *α* set at 0.05. Each clinical center treated 8 patients for a total of 48 (96 implants; 48 test, 48 control) to compensate eventual drop-outs occurring during the follow-up period.

### 2.6. Randomization

A table was prepared by using a web-based software (www.randomization.com) with a balanced, randomly permuted block approach, distributing first and second site of each patient into two groups (test=UISP; control=drills). The randomization codes were enclosed in numbered, sealed, opaque envelopes which were opened by a clinical assistant after flap elevation in the first site. Treatment allocation was then concealed to the surgeon in charge of recruiting and treating the patients included in this clinical trial.

### 2.7. Statistical Analysis

Statistical analysis was performed by using a statistical software package (SPSS 22.0, SPSS Inc., Germany). Parametric methods were used for all the datasets. Data normality (with the exception of age) was assured by root square transformation, as assessed through the Kolmogorov-Smirnov test. However, mean and standard deviations of nontransformed data were used for descriptive purposes. The significance of the difference in surgical time between the two groups was assessed through a paired sample t-test [36]. The significance of the difference in marginal bone loss between the groups within each time point and between the time points within each group was assessed by a paired sample t-test.

Finally, for each group, a forward multiple linear regression was used to evaluate the association between marginal bone loss at 12 months (dependent variable) and other independent variables (age, gender, smoking habits, history of periodontal disease, and implant insertion area). The cut-off levels of significance used were 0.05 and 0.10 for entry and removal, respectively.

A p value <0.05 was considered statistically significant.

## 3. Results

Forty-eight patients were enrolled, randomized, and treated with the insertion of two implants: each clinical center contributed with 8 patients. Eight patients dropped out from the study: eight implants in four patients did not reach a sufficient primary stability to be immediately loaded (35 Ncm) and were submerged under the soft tissues (four in test group; four in control group; no difference between groups); four implants in four patients failed within the first six months of healing (two in test group; two in control group; no difference between groups; 4.5% cumulative failure rate). No additional implants were lost and no other biological or mechanical complications were recorded during the first two years after implant positioning.

Forty patients (age 60.1±10.7 years; range 39–79 years; 22 female, 18 male) with eighty implants (40 test; 40 control) were included in this study. Twenty-six patients referred to be no smokers, eleven light smokers, and three heavy smokers. Main demographic characteristics are summarized in Table 1. Mean MBL of the entire sample after six months of loading was 1.41±1.09 mm, after one year was 2.03±1.36 mm, and after two years was 2.11±1.07 mm. Mean MBL after six months of loading in the test group was 1.39±1.03mm, after one year was 1.92±1.14 mm, and after two years was 1.95±0.99 mm. Mean MBL after six months of loading in the control group was 1.42±1.16mm, after one year was 2.14±1.55 mm, and after two years was 2.22±1.04 mm. Differences in marginal bone loss within test and control groups at 6 months and one year was statistically significant (p<0.0001). Differences between test and control group at six months and one year were not statistically significant (p>0.05).

Mean surgical time was 395.2±171.3 sec (range 120-810 sec) in the test group and 304.6±148.0 sec (range 120-600 sec) in the control group: difference between the two groups was statistically significant (p=0.001).

Multiple linear regression analysis did not demonstrate a significant association between marginal bone loss and any of the explanatory variables (age, gender, smoking habits, history of periodontal disease, and implant insertion area; data not shown).

## 4. Discussion

UISP was previously clinically tested on a large number of patients showing that this novel approach could represent a reliable alternative to traditional drilling protocols [37, 38]. Recent studies showed that UISP leads to a limited decrease of primary stability during the early phases of bone healing [32–34], likely due to a slightly different biochemical response in the osteotomy area. In particular, researchers focused on the receptor activator of nuclear factor kappa-B-ligand (RANKL) and osteoprotegerin molecular system, which control the cellular cascade regulating bone resorption process [39]. A recent human study demonstrated lower RANKL levels in implant sites prepared by ultrasonic devices compared to sites prepared by using traditional drilling systems, suggesting decreased osteoclastic activity [40].

On these premises, the present trial was specifically designed to assess the clinical impact of different implant site preparation techniques on the outcomes of immediately loaded single implants.

Eight implants in four patients did not reach an insertion torque ≥35 Ncm and were not immediately loaded, dropping out from the study: these implants were all inserted in low quality maxillary bone where both test and control site preparation technique failed in reaching a sufficient primary stability. This is in accordance with the studies by Baker et al. [41] and Gandhi et al. [42], demonstrating that UISP affords similar primary implant stability in comparison to conventional rotary instrumentation.

Four implants in four patients failed during the first six months of healing and no other implants were lost at two-year follow-up: cumulative survival rate was 95.5%, which is an acceptable result considering that a recent meta-analysis by Sanz et al. (2015) stated that immediately loaded single implants are at greater risk of failure, when compared to immediately loaded bridges or full arch restorations [43]. However, no difference was found between the two arms of the present study (2 failed implants in both groups).

The traditional drilling protocol required a lower operative time than UISP: difference in surgical time between the two groups reached statistical significance in the present trial (p=0.001). This finding is in accordance with all previously published studies comparing the two techniques in terms of duration of the intervention [32, 40, 44]; however, even if the difference was statistically significant, it could be considered clinically irrelevant if balanced by surgical or biological advantages.

Mean interproximal MBL was 1.41 mm after 6 months and 2.03 mm after one year: even if implants inserted with UISP technique resulted in a slightly lower MBL than the control group (1.39 and 1.42 mm at six months; 1.92 and 2.14 mm at one year, respectively), no statistically significant differences were demonstrated between the two groups. Between one-year and two-year follow-up, no statistically significant difference in MBL was demonstrated in both groups, suggesting a stabilization of marginal bone levels. The multivariate analysis did not show a significant influence of patient-related variables (age, gender, smoke, history of periodontitis, and implant insertion site) on MBL.

However, in the present study both test and control groups resulted in a greater mean MBL if compared to data present in literature: seven RCT included in a recent systematic review on immediately loaded single implants reported MBL ranging from 0.24 to 0.91 mm at one-year follow-up [6].

Numerous variables, including patient habits [45], surgical technique [46], soft tissue and alveolar bone thickness [47–49], implant and abutment design (e.g., macro- and microgeometry, connection characteristics, implant crest module, and abutment height) [50–53], number of abutment disconnections [54], and prosthetic features (e.g., screwed versus cemented retention, inadequate occlusion) [55, 56] have been identified as influencing factors in the genesis of peri-implant bone resorption. However, due to their simultaneous action, the exact role and importance of each factor, together with their complex interactions, are not completely clarified yet [57]. In the present study, most of these confounding factors have been controlled by inclusion and exclusion criteria, in order to evaluate the effect of implant site preparation technique. Therefore, the analysis of the factors causing MBL in this trial should be focused on two main factors: implant crest module and characteristics of provisional abutment. Some authors demonstrated that a parallel-sided implant crest module with smooth surface results in a greater shear stress in the crestal region than an angled crest module with rough surface, increasing the risk of marginal bone resorption [50, 51, 58]. Moreover, other studies showed that the presence of microthreads in the implant neck could provide a positive contribution to bone implant contact and to the preservation of the marginal bone [59–61]. The implant used in this study had a parallel-sided crest module with a polished collar without microthreads: these features could have favoured the transmission to the crestal bone of a greater amount of shear forces in comparison to compressive and tensile components. The detrimental role of this force distribution could have a particularly negative influence on marginal bone stability in immediate loading conditions: in fact, a previous study using the same implants but applying a delayed loading (3 months after insertion) reported a mean MBL of 0.8 mm at three-year follow-up [62].

**Figure 4:**
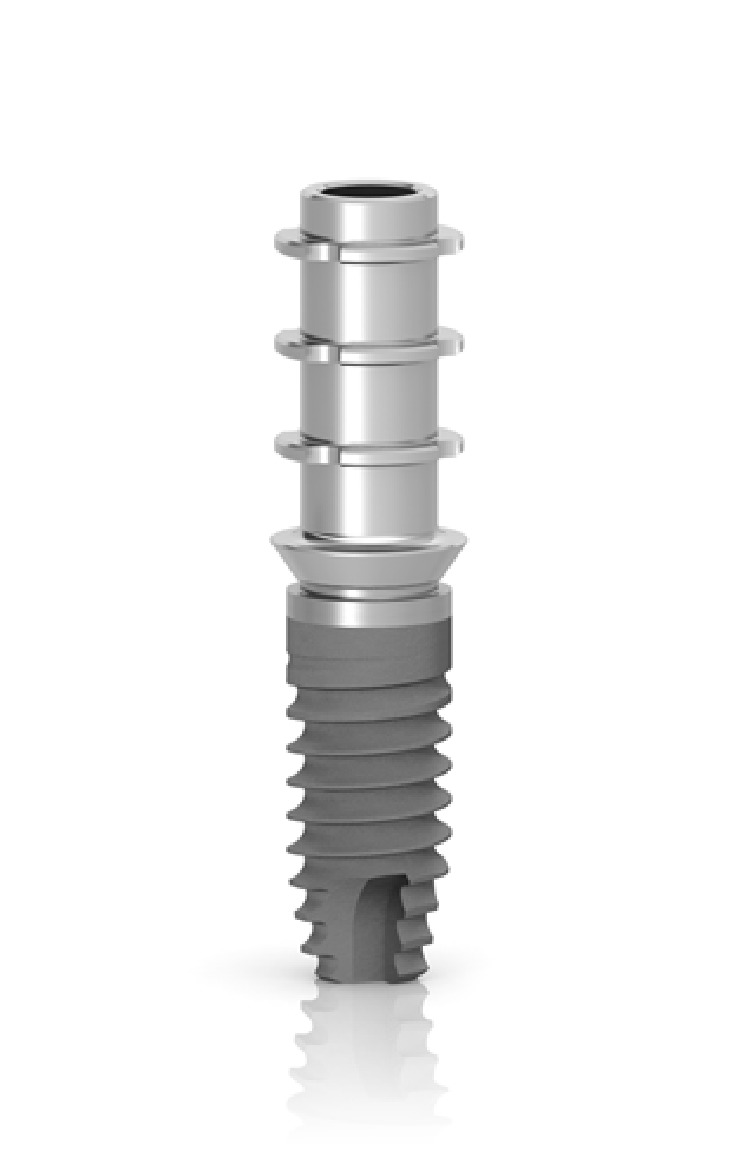
The provisional abutment used in this study presented 1.5 mm height from the implant platform.

The second factor to be evaluated for its contribution to MBL is the height of the provisional abutment. Numerous authors demonstrated a strict relationship between prosthetic abutment height and peri-implant bone loss, possibly due to a reestablishment of the biological width [63–66]: in particular, Galindo-Moreno and coworkers suggested that a prosthetic abutment height <2 mm is significantly related to higher MBL rates than longer abutments, irrespective of the presence of a platform-switched connection [53]. The height of the provisional abutment used in this study was 1.5 mm (Figure 4): this factor could also have contributed to promote marginal bone resorption during the healing period.

The possible negative impact of the selected implant-abutment system on the maintenance of marginal bone level represented the major limitation of this study. Other possible limitations were represented by the relatively low number of patients and by the partial standardization of bone quality within the single patient. The number of patients included in the final analysis was sufficient to satisfy the minimum sample size requirements of the study, but trials on a broader population are recommended. Moreover, a split-mouth design should be considered, in order to minimize variability between test and control site in terms of bone quality.

Therefore, the aforementioned findings allowed us to accept the null hypothesis of the study of no differences in survival rate and marginal bone loss between UISP (test group) and conventional site preparation with rotary instruments (control group) in immediately loaded dental implants: UISP might be used as a reliable alternative to the traditional drilling systems, coupling similar clinical outcomes with the characteristics of enhanced surgical control and safety in proximity of delicate structures.

Future research should focus on long-term follow-up to determine both implant- and patient-based outcomes of UISP. Comparative clinical trials with split-mouth design in different clinical situations (delayed loading, immediate loading, single implant, and multiple implants) should be designed and conducted.

## 5. Conclusions

Within the limitations of this study, UISP for immediately loaded single implants resulted in similar clinical outcomes (implant survival rate and marginal bone loss), when compared to conventional rotary instrumentation. Further clinical trials on greater samples and additional long-term studies are necessary to confirm these findings and completely understand the possible clinical advantages of bone healing process after ultrasonic surgery.

## Figures and Tables

**Table 1:** 

Demographic characteristics	
Male	18 (45%)
Female	22 (55%)
Mean age (range)	60.1 (39-79)
No smoker	26 (65%)
Light smoker (<10)	11 (27.5%)
Heavy smoker (≥10)	3 (7.5%)

## Data Availability

The datasets generated and analyzed during the current study are available from the corresponding author upon reasonable request.
